#  Gastrointestinal Stromal Tumor: Cause of Gastrointestinal Bleeding

**DOI:** 10.4021/gr2010.03.178w

**Published:** 2010-03-20

**Authors:** Ramon Vilallonga, Jose Luis Sanchez, Manel Armengol

**Affiliations:** a Department of Surgery, General Universitary Hospital Vall d'Hebron, Autonomous University of Barcelona, Passeig de la Vall d’Hebron, 119-129, 08035 Barcelona, Spain

**Keywords:** Gastrointestinal stromal tumor, Gastrointestinal Bleeding, surgery

## Abstract

Gastrointestinal stromal tumors (GISTs) are rare tumors and can be a cause of gastrointestibnal bleeding, when other causes have already been excluded. This mesenchymal tumors can be diagnosed hardly, and they should be included in any differential diagnosis. Our case illustrates the difficulty of diagnosis and sometimes shows how non-invasive test can be few helpful. Surgery is very often indicated, and becomes therapeutic and diagnostic. GIST tumors are rare and surgical resection with curative intent is the treatment of choice.

## Introduction

Gastrointestinal stromal tumors (GISTs) are uncommon and can be a cause of obscure hemorrage when conventional investigations such as esophagogastroduodenoscopy and colonoscopy fail to detect bleeding lesions. GIST tumors, a rare group of neoplasias of the gastrointestinal tract (GI) and considered to be cause of obscure GI bleeding, have small intestinal lesions in 27% of the patients [1].

GISTs are mesenchymal tumors specific for the GI tract (60% in stomach, 30% small intestine, 10% elsewhere). From 10 to 30% of them are malignant and show liver metastases or intra-abdominal spread, they represent only 3% of all malignant GI tumors [2].

The first series of GISTs were reported by Golden and Stout [3]. The digestive hemorrhage occurs very often because they often grow in an extraluminal direction and if they erode or ulcerate through the small bowel mucosa they can cause intermittent bleeding. The abdominal pain, abdominal mass, obstruction or the perforations are the main symptoms [4].

Diagnosing these lesions is difficult because they tend to be inaccessible to routine endoscopy, like in our case. Variable sensitivities and specificities for diagnosing small intestinal lesions are found with small bowel barium studies, selective visceral angiography, wireless capsule endoscopy, radioactive isotope bleeding scans and exploratory laparotomy is often the last option [5, 6]. We do present our experience in a patient with a gastrointestinal stromal tumor with bleeding.

## Case Report

A 77-year-old woman presented with 3 days of hematochezia associated with mild lower abdominal. The patient had hypertension, obesity and in the past medical history was a history of a bleeding in 2 occasions, requiring blood transfusion, and attributed to gastric ulcers treated.

Physical examination on admission revealed conjunctival pallor. Workup included an esophagogastroduodenoscopy with negative results, a colonoscopy showing blood throughout the colon, diverticulosis and fresh bleeding from the terminal ileum.

**Figure 1:**
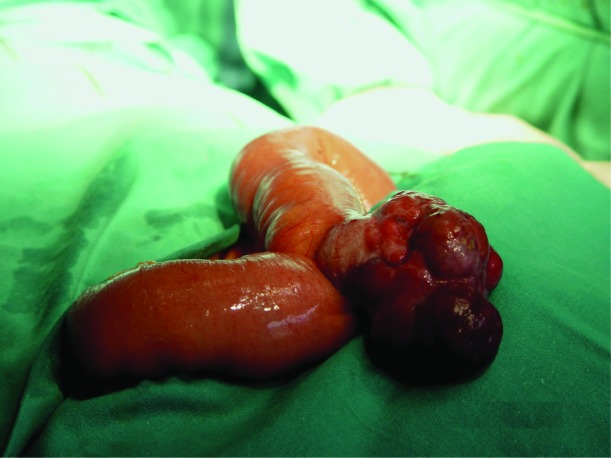
Jejunal highly vascular mass on the antimesenteric border.

A computed tomography scan was performed and showed a 4 x 4 cm mass in the ileal area. A celiac and superior mesenteric artery angiogram revealed a highly vascular midjejunal mass on the antimesenteric border of the bowel. A laparotomy was performed and the tumor was excised (Fig. 1) and identified histologically as a gastrointestinal stromal tumor (GIST), with c-Kit (CD117 and CD34) markers positive. The patient recovered postoperatively without incidences and is clinically stable at 3 years postresection, without evidence of recurrent disease on follow-up CT scans.

## Discussion

Reaching the diagnostic of small intestine hemorrhage is difficult, and in as many as 5% of patients with obscure GI bleeding, a source cannot be identified despite extensive examination [7]. In many occasions, lesions cannot be identified after upper endoscopy and colonoscopy, and more specific studies must be performed to explore the small intestine such as enteroclysis, sonde enteroscopy, wireless capsule endoscopy or even intraoperative enteroscopy [8].

Tumors and vascular ectasias are the lesions most commonly identified as bleeding sites in the small bowel in old patients. In patients with less than 25 years of age, Meckel’s diverticulum is the most common source of small bowel bleeding [8].

Some other rare causes of GI hemorrhage include hemobilia, Dieulafoy lesion, aortoenteric fistula, extraesophageal varices, and diverticula. GIST should be considered in the differential diagnosis for GI bleeding. These tumors, in 50% of the cases, presented with subacute or acute GI bleeding [9].

GISTs are the most common mesenchymal tumors of the stomach and small intestine [10]. They possess unique histologic, immunophenotypic, and molecular genetic features that set them apart from typical leiomyomas and schwannomas [10]. GIST’s immunohistochemical staining is usally positive for CD117 (c-Kit protein) and CD34 (hematopoietic cell progenitor cell antigen), like in our case [11].

GISTs are typically discovered incidentally during endoscopic, radiologic, or surgical procedures or are diagnosed in the evaluation of patients presenting with an abdominal mass, abdominal pain, or GI bleeding.

Surgical resection with curative intent is the treatment of choice with localized GIST [12]. Complete resection of the tumor should be always performed, because recurrence can appear. The extent of surgical excision and survival has no relationship [9]. If feasible limited resection is possible, those should be performed, because an adequate extended resection provides no survival benefit over wide local excision [9].

Approximately 10% of all GISTs display malignant behaviour [10]. When the maximal diameter of the GISTs is superior to 10 cm, malignancy should be considered. Furthermore, this tumor shows mucosal ulceration, necrotic areas and hemorrhage [10]. Cytologic atypia is not always seen, and this makes more difficult the diagnosis.

Concerning the hemorrhagic potential of this tumor, some authors have already reported high incidences of presenting with bleeding, 87% of duodenal GISTs and 64% of other small bowel GISTs. Other locations like gastric, rectal or colonic are associated with less than 45% incidence of bleeding [12].

In conclusion, GI bleeding in patients can be caused by GISTs tumors. Even if rare, they must be included in the differential diagnosis. Treatment is still complete surgical resection.
